# Preserved neurogenesis in non-demented individuals with AD neuropathology

**DOI:** 10.1038/srep27812

**Published:** 2016-06-14

**Authors:** David Briley, Valeria Ghirardi, Randy Woltjer, Alicia Renck, Olga Zolochevska, Giulio Taglialatela, Maria-Adelaide Micci

**Affiliations:** 1 Mitchell Center for Neurodegenerative diseases, Department of Neurology, University of Texas Medical Branch, Galveston, TX, USA; 2 Department of Pathology, Oregon Health & Science University, OR, USA; 3 Department of Anesthesiology, University of Texas Medical Branch, Galveston, TX, USA.

## Abstract

Rare individuals remain cognitively intact despite the presence of neuropathology usually associated with fully symptomatic Alzheimer’s disease (AD), which we refer to as Non-Demented with Alzheimer’s disease Neuropathology (NDAN). Understanding the involved mechanism(s) of their cognitive resistance may reveal novel strategies to treat AD-related dementia. In the pursuit of this goal, we determined the number of hippocampal neural stem cells (NSCs) and investigated the expression of several miRNAs in NDAN and AD subjects. Laser-capture microdissection of autopsy human hippocampus DG and qRT-PCR miRNA analyses were combined with immunofluorescence in this study. The number of SOX2^+^ NSCs in the DG was significantly increased in NDAN individuals as compared to AD subjects. Further, the prevalence of SOX2^+^ NSCs was found to correlate with cognitive capacity. Neurogenesis-regulating miRNAs were decreased in NDAN individuals as compared to AD patients. An increased number of NSCs and new neurons in NDAN individuals is associated with a unique expression of regulating miRNAs and strongly support a role of neurogenesis in mediating, in part, the ability of these individuals to resist the pathological burden of AD.

Despite extensive research effort on Alzheimer’s disease (AD) now being into its third decade^1^, exactly how AD starts, how the disease progresses, and how to stop or to slow its progression are still all unresolved questions^2^. A handful of pharmacological interventions are available and approved as palliative treatments, but these show only limited efficacy in a small population of patients, and for only a limited time^3^.

In recent years several reports have described rare individuals who remain cognitively intact despite the presence of neuropathological features usually associated with a fully symptomatic stage of AD^4^,^5^,^6^,^7^,^8^,^9^. The existence of these unusual cases, herein referred to as Non-Demented with Alzheimer’s disease Neuropathology (NDAN), suggests that there is a natural way for the human brain to resist (or significantly delay) the neurotoxic events that normally lead to cognitive impairment in AD. Understanding the mechanisms involved in such cognitive resilience may suggest conceptually novel treatment strategies for AD centered on promoting in affected individuals endogenous resistance to disease-driven cognitive decline.

The discovery that new neurons are continuously generated in the hippocampus, an area of the brain that plays a critical role in learning and memory and is most affected by AD^10^, suggests that plasticity of the central nervous system could provide an endogenous protective mechanism to sustain cognitive functions. Indeed a growing body of literature has emerged demonstrating a strong correlation between neurogenesis, memory and cognitive function in animal models. Notably, promotion of adult hippocampal neurogenesis is associated with improved spatial memory, while a decline in neurogenesis underlies cognitive impairments reportedly associated with aging, trauma and various neurodegenerative disorders including AD^11^,^12^,^13^,^14^,^15^.

The process of neurogenesis in the hippocampus consists of several phases, each one corresponding to different stages of maturation of the developing cells. Type 1 neural stem cells (NSCs) generate neuroblasts and immature granule cells, which in turn differentiate into mature granule cells. SOX2 is a transcription factor that has been shown to play a critical role in the maintenance of stem cell pluripotency and is commonly used as a marker of NSCs in the dentate gyrus of the hippocampus^16^,^17^,^18^,^19^.

Adult hippocampal neurogenesis is modulated by a variety of genetic and epigenetic factors^20^,^21^. In addition, because the differentiation and maturation of newborn neurons involves the concerted action of multiple genes, micro-RNAs (miRNA), short non-coding RNA sequences that bind to mRNA targets and inhibit their translation, have been recently identified as important regulators of neurogenesis^22^,^23^.

To ask whether neurogenesis is linked to preserved cognitive ability in humans with AD neuropathology, in this study we evaluated the expression of SOX2 and of the mature neuronal marker NeuN in post-mortem human tissues from NDAN, mild cognitively impaired (MCI) and AD individuals in comparison to age-matched healthy subjects. In order to begin investigating the mechanisms involved in the regulation of neurogenesis in demented and non-demented individuals, we further analyzed the expression of selected microRNAs in laser-captured dentate gyrus samples from autopsy specimens of NDAN, MCI, AD and age matched healthy subjects.

## Results

### SOX2 and NeuN are co-expressed in the human hippocampus dentate gyrus

**Figure 1:**
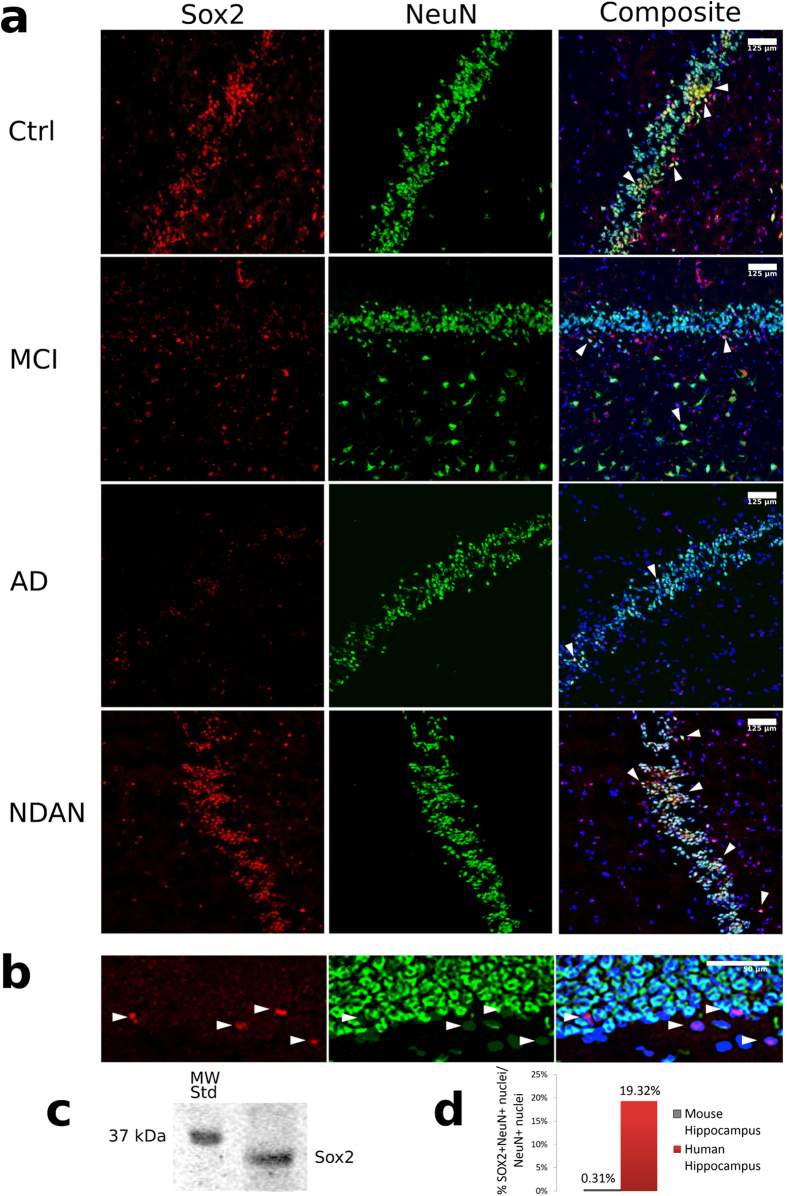
SOX2 and NeuN are co-expressed in the human dentate gyrus. (**a**) Representative images of human hippocampus sections showing immunofluorescence staining for SOX2 (red) and NeuN (green) in the dentate gyrus. Nuclei are counterstained blue with DAPI. Arrowheads indicate cells that co-express SOX2 and NeuN. (**b**) Immunofluorescent staining of SOX2 (red) and NeuN (green) in wild type mouse hippocampus dentate gyrus. Nuclei are stained blue with DAPI. (**c**) Representative Western blot showing human hippocampus lysate probed with the same anti-SOX2 antibody used in A and B. A band corresponding to the predicted molecular weight for SOX2 (34 kDa) is detected. (**d**) Flow cytometry analysis of NeuN and SOX2 expression in nuclei isolated from mouse and human hippocampus. Data is expressed as percentage of nuclei co-expressing SOX2 and NeuN versus total neuronal nuclei (expressing NeuN).

SOX2 immunoreactivity was observed in both the granular cell layer (GCL) and subgranular zone (SGZ) of the dentate gyrus (DG) in all autopsy human hippocampus specimens analyzed. In some cells, the expression of SOX2 co-localized with the neuronal marker NeuN (Fig. 1). This was surprising because, in the rodent DG, SOX2 expression is limited to undifferentiated NSCs and is not observed in NeuN^+^ granule cells^19^. We therefore tested the specificity of our antibody by performing immunofluorescence staining of the murine hippocampus (Fig. 1b) and by Western blot analysis of human hippocampus total protein lysate (Fig. 1c). The results confirmed that the antibody used specifically recognizes SOX2 and that, contrary to the human DG, in the murine DG, SOX2 did not co-localize with NeuN^+^ nuclei. Same results were obtained when nuclei extracted from frozen human or mouse hippocampus were analyzed by flow cytometry, according to methods published earlier^24^, after co-staining with SOX2 and NeuN. Nuclei from human hippocampus showed a substantial co-localization of SOX2 and NeuN (19.32% of the NeuN-expressing nuclei), whereas nuclei from mouse hippocampus showed virtually none (Fig. 1d). Thus, the observed co-localization of SOX2 and NeuN in the human DG appears to be specific. An attractive possibility is that such SOX2/NeuN co-localization in the human DG may reflect newly formed neurons where SOX2 expression has not yet been turned off.

### SOX2^+^ cells are increased in the hippocampus of NDAN individuals

**Figure 2:**
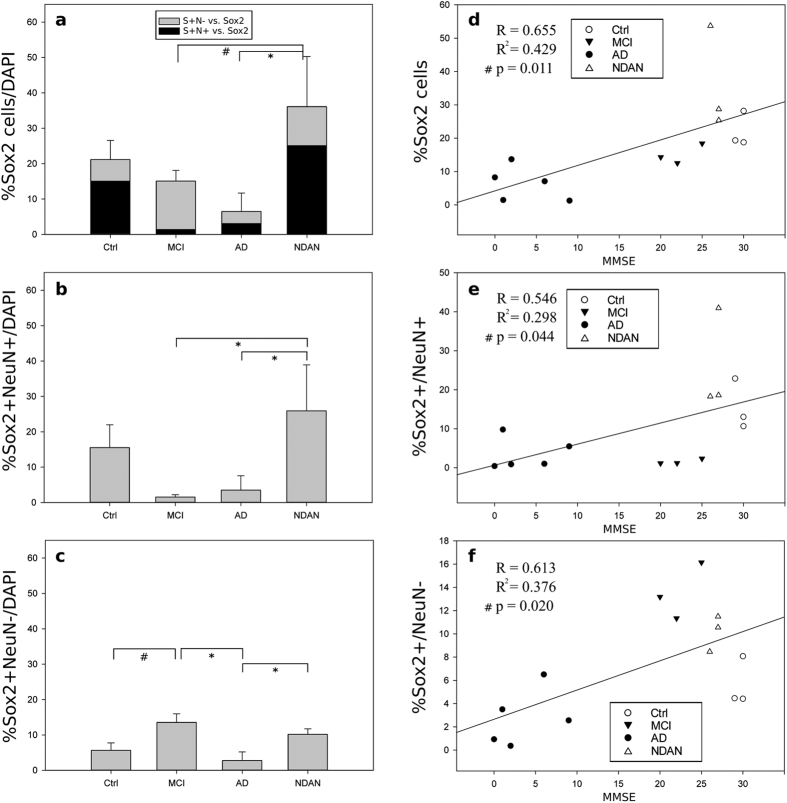
Quantification of SOX2 expression in the human dentate gyrus. The number of cells immunoreactive for SOX2 and NeuN in human dentate gyrus specimens was quantified by an independent investigator who was blinded to the experimental groups and normalized to the number of DAPI^+^ nuclei. Colocalization of SOX2 and NeuN was assessed using ImageJ plugins. Values are represented as the mean percentage of positive cells/total nuclei ± SEM. (**a**) The percentage of all SOX2^+^ cells (inclusive of NeuN^+^ and NeuN^−^ cells) is significantly increased in NDAN as compared to both MCI and AD. Within each column the percentage of SOX2^+^ cells that are negative for NeuN (grey) or positive for NeuN (black) is shown. (**b**) The percentage of cells that are positive for both SOX2 and NeuN (SOX2^+^/NeuN^+^) is significantly greater in NDAN as compared to MCI and AD. (**c**) The percentage of cells that express SOX2 but are negative for NeuN (SOX2^+^/NeuN^−^) are significantly increased in MCI as compared to AD and control and significantly increased in NDAN as compared to AD. Statistical significance was determined by ANOVA followed by multiple comparison procedures using the Holm-Sidak method. ^#^p < 0.05; *p < 0.01. Linear regression analysis was performed to examine the relation between the percentage of SOX2^+^ cells (**d**), SOX2^+^/NeuN^+^ cells (**e**), SOX2^+^/NeuN^−^ cells (**f**) and cognitive function (MMSE score). Positive and significant correlations were found for all the regression performed, p < 0.05.

The total number of SOX2^+^ cells in the DG was significantly increased in NDAN individuals as compared to both MCI and AD patients (Fig. 2a). There was also a trend of a higher number of SOX2^+^ cells in NDAN as compared to age-matched controls that however did not reach statistical significance. When we quantified both the number of SOX2^+^/NeuN^+^ cells and of SOX2^+^/NeuN^−^ cells, the percentage of SOX2^+^ cells also expressing NeuN was increased in NDAN individuals while it was decreased in both MCI and AD patients as compared to age-matched healthy controls (Fig. 2b). On the other hand, the percentage of cells expressing only SOX2 (SOX2^+^/NeuN^−^) was increased in both NDAN and MCI subjects, while it was decreased in AD patients as compared to age-matched healthy controls (Fig. 2c).

**Figure 3:**
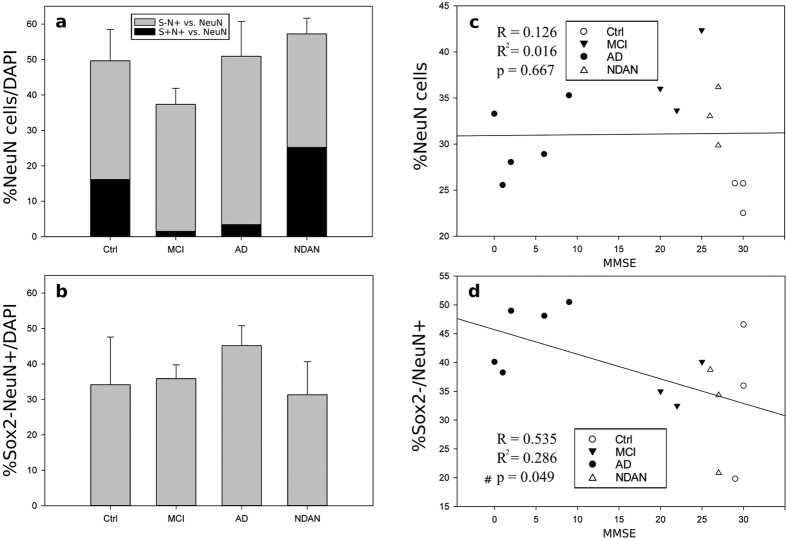
Quantification of NeuN expression in the human dentate gyrus. (**a**) No significant differences between the groups are observed in the percentage of NeuN positive cells as a proportion of DAPI-positive cells. Within each column, the percentage of NeuN^+^ cells that are negative for SOX2 (grey) or positive for SOX2 (black) is shown. (**b**) No differences between the groups are observed in the percentages of cells expressing NeuN but not SOX2 (NeuN^+^/SOX2^−^). (**c**) Linear regression analysis show no correlation between the percentage of total NeuN^+^ cells and cognitive function (MMSE score), while (**d**) it showed a significant negative correlation between the percentage of NeuN^+^/SOX2^−^ cells and MMSE, p < 0.05.

In order to determine whether the number of granular neurons differed between demented and non-demented individuals with AD pathology, we quantified the number of cells expressing only NeuN. The total number of NeuN^+^ cells was not significantly different between NDAN, MCI, AD and age-matched control subjects (Fig. 3a). Similarly, no differences were found in the percentage of cells expressing NeuN but not SOX2 (SOX2^−^/NeuN^+^), and presumed to be mature neurons (Fig. 3b).

### The number of SOX2^+^ cells in the DG correlates with cognitive function

A linear regression analysis was performed to correlate the mini mental state examination (MMSE) scores of each individual examined with the proportion of cells expressing SOX2 and NeuN. A positive correlation was found between MMSE scores and the number of SOX2^+^ cells (Fig. 2d). Similarly, a positive correlation was found between MMSE scores and the number of SOX2^+^/NeuN^+^ cells (Fig. 2e), and the number of SOX2^+^/NeuN^−^ cells (Fig. 2f). Conversely, MMSE scores did not correlate with the total number of cells expressing NeuN (Fig. 3c) and a negative correlation was found between the number of NeuN^+^/SOX2^−^ cells and MMSE scores (Fig. 3d).

In order to control for possible confounding effects on protein expression due to variability in tissue collection time, we performed a correlation analysis between the postmortem interval of tissue collection and SOX2 or NeuN expression. The results showed that the postmortem interval did not correlate with any of the measurements made (Supplementary Figure 1).

### Levels of miRNAs modulating neurogenesis differ between non-demented (NDAN) and demented (MCI and AD) individuals

**Figure 4:**
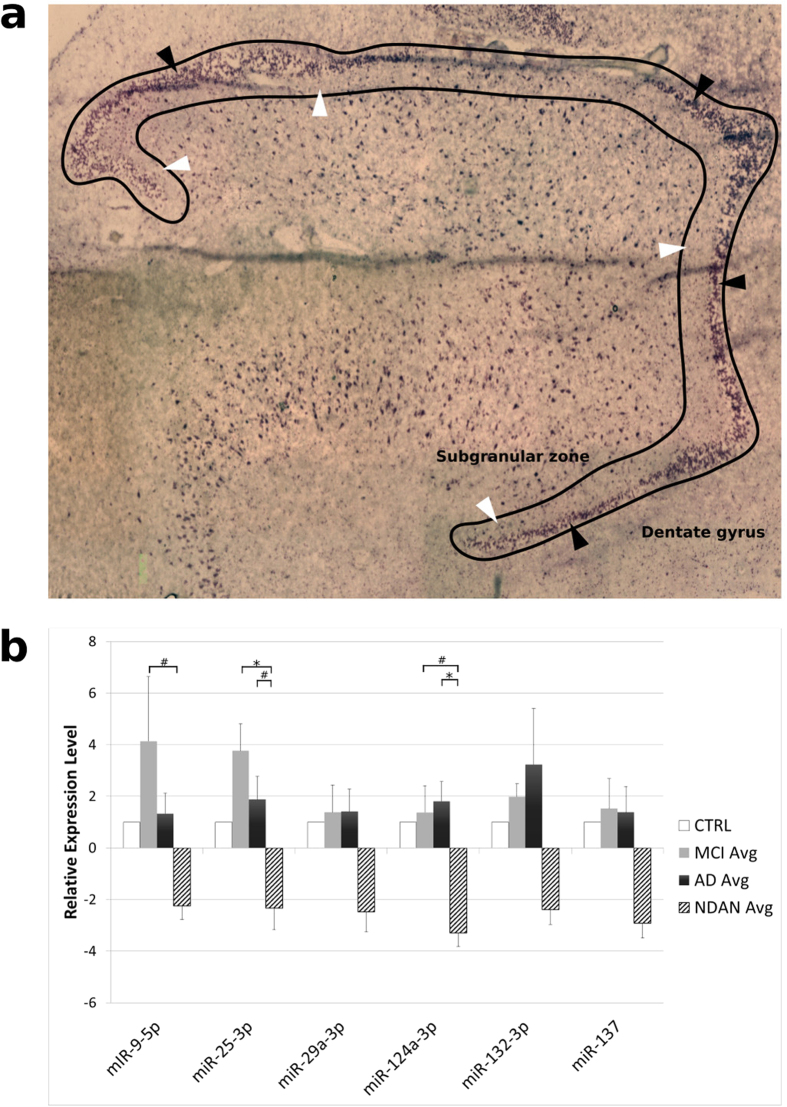
The expression of selected miRNAs in the DG is significantly different between non-demented and demented individuals with AD pathology. (**a**) Representative section of human DG stained with cresyl violet. The black outline indicates the region collected by laser capture microdissection (LCM). Black arrowheads (▲) indicate the granular cell layer (GCL). White arrowheads indicate the subgranular zone (SGZ). (**b**) The relative level of expression of six miRNAs, known to play a role in the regulation of neurogenesis, was determined in the DG by qRT-PCR and fold changes were calculated using the 2^−ΔΔCt^ method. Age-matched healthy controls were used as calibrator, with its fold change taken as 1. The relative expression of all evaluated miRNAs between groups was found to be significantly less in NDAN than both MCI (Q = 1.507, p < 0.001) and AD patients (Q = 5.247, p < 0.001; One Way ANOVA). For each miRNA, statistical analysis was performed by one-way ANOVA followed by pairwise multiple comparison procedures using the Holm-Sidak method, #p <0.05, *p <0.01.

We used qRT-PCR to measure the levels of selected miRNAs known to regulate all stages of neurogenesis (miR-9, miR-25, miR-29a, miR-124, miR-132, miR-137)^23^,^25^,^26^ in laser-captured DG samples from the NDAN, MCI, AD and healthy controls (Fig. 4a). We found that the levels of miR-9, miR-25 and miR-124 were significantly lower in NDAN subjects as compared to MCI individuals and miR-25 and miR-124 lower as compared to AD patients (Fig. 4b). The same pattern of lower levels in NDAN subjects was observed for miR-29, miR-132 and miR137, which however did not reach statistical significance (Fig. 4b). Figure 4b also shows that, regardless of individual statistical significance, the levels of all miRNAs assayed followed a pattern of reduction in NDAN, whereas there was a consistent pattern of increased miRNAs levels in MCI or AD. When we considered all different miRNAs in aggregate for each patient group, we found that there was a highly significant difference between NDAN subjects and both MCI and AD patients, confirming a significant pattern of decreased levels for all measured miRNAs in NDAN contrasted by a pattern of increased levels in MCI or AD patients. These data strongly suggest that in NDAN there may be an epigenetic regulation of neurogenesis driven by lower levels of modulating miRNAs.

## Discussion

NDAN individuals are characterized by normal cognition at the time of their death, despite the presence of amyloid plaques and neurofibrillary tangles characteristic of a fully developed disease. The aim of this work was to study neurogenesis in the hippocampus DG of NDAN and to compare it to MCI subjects, AD patients and healthy age-matched control individuals. Because it is not possible to measure neurogenesis as a dynamic process in the human brain, due to the practical inability to track newborn cells and follow their differentiation over time, we used the expression of SOX2, a key regulator of neural stem cells^18^,^19^, as an indicator of neurogenic potential in human hippocampus samples.

Firstly, we found that in the human DG, the expression of SOX2 overlaps with the neuronal marker NeuN in a subset of cells. This was surprising because in the rodent DG the expression of SOX2 is restricted to undifferentiated NSCs^19^,^27^. Indeed, using the same antibodies utilized in the human brain samples, we confirmed that SOX2 and NeuN are not co-expressed in the murine DG. Furthermore, flow cytometry analysis of nuclei isolated from human or mouse hippocampi also confirmed co-localization of SOX2 and NeuN in human but not in mouse specimens. We conclude therefore that in the human DG, SOX2 expression persists for a longer period of time during the differentiation of neural progenitor cells, and that SOX2^+^/NeuN^+^ cells could represent newly formed neuronal cells.

Consistently with previous reports^28^, we found fewer SOX2^+^ cells in the DG of AD patients as compared to healthy controls. On the other hand, in NDAN individuals, SOX2^+^ cells were significantly more abundant in the DG as compared to both AD and MCI subjects and even higher than healthy subjects.

Interestingly, when we analyzed the proportion of cells co-expressing SOX2 and NeuN, we found that these were significantly more abundant in NDAN DG as compared to both MCI and AD subjects, whereas, on the other hand, cells that exclusively express SOX2 (NeuN^−^) were significantly more abundant in both NDAN and MCI DG as compared to AD and healthy controls. These data suggest that not only do NDAN individuals have a greater number of SOX2^+^ cells in the DG, but also that these cells possess a greater capacity to generate new neurons. On the other hand, SOX2^+^ cells in MCI individuals remain mostly in the undifferentiated state with the majority of cells not expressing NeuN. Linear regression analysis confirmed that SOX2 expression, whether co-expressed with NeuN or alone, positively correlates with preserved cognitive function. Specifically, higher percentages of both SoX2^+^/NeuN^−^ cells, corresponding to undifferentiated NSC, and SOX2^+^/NeuN^+^ cells, likely representing newly formed neurons, significantly correlate with higher MMSE scores.

The changes in the percentages of SOX2^+^ cells in the DG cannot be explained by a reduced number of granular neurons because the overall expression of NeuN showed no significant differences among the groups, indicating that the total number of granular neurons in the DG remain constant. Interestingly, a negative correlation was found between cells expressing only NeuN (Sox2^−^) and MMSE scores, thus suggesting that these cells might represent an older population of neurons and that having more of these older neurons is associated with poor cognitive performance.

Recent evidence has identified small non-coding RNAs (miRNAs) as important regulators of neurogenesis^23^. Here, we measured the expression of six miRNAs known to modulate neurogenesis in the DG, and found that the levels of all miRNAs assayed were approximately two-fold lower than control subjects in NDAN, whereas they were consistently higher than control in MCI and AD DG. Although, when analyzed individually, only miR-9, miR-25 and miR-124 reached statistical significance, when considered as a group, the level of all miRNAs analyzed was highly significantly different between NDAN and both MCI and AD, showing a consistent pattern of decrease in the former one and of increase in the latter two. Because miRNAs can affect a wide array of targets, it is difficult to determine the influence of each individual miRNA analyzed in our study on human DG neurogenesis (including proliferation, migration and differentiation of NSCs). However, our data demonstrate that the levels of such miRNAs in aggregate show a distinctive pattern that sets NDAN apart from both cognitively affected groups (MCI and AD), further supporting our results showing both increased number of NSCs in NDAN and of their neurogenic potential. Thus, in NDAN a unique epigenetic regulation mediated by miRNAs could be responsible for sustained levels of NSCs (SOX2^+^/NeuN^−^) and their ability to generate new neurons (SOX2^+^/NeuN^+^ ) in the DG.

Interestingly, we found that both the number of SOX2^+^ cells and the level of regulating microRNAs (specifically miR9 and miR25) were much higher in the DG of MCI subjects as compared to both AD and healthy individuals. This suggests that in the early phases preceding a fully symptomatic AD, increased proliferation of NSC in the DG might represent an attempt of the brain to counteract the progression of the disease. It is interesting to note however that, while the number of SOX2^+^ cells is increased in MCI individuals, the number of SOX2^+^ cells that co-express NeuN is decreased. This observation, along with the concomitant increased levels of specific regulating microRNAs, suggests that the neurogenic potential of NSC is impaired even at an early stage of AD progression.

Several published reports have shown a correlation between the rate of neurogenesis and brain function in rodents^29^. Specifically, decreased proliferation of NSC in the DG results in impairments in learning and memory, while on the other hand, external factors known to increase NSCs proliferation and neurogenesis, like exercise and environmental enrichment, result in improved learning and memory^29^,^30^,^31^. It is therefore tempting to speculate that sustained neurogenesis in the DG of NDAN subjects is an important factor mediating their ability to evade dementia in spite of the presence of a degree of neuropathology (plaques and tangles) usually associated with clinically manifest AD.

In conclusion, our data strongly suggest that NDAN individuals have increased neurogenesis in the DG, likely driven by unique changes in the levels of modulating miRNAs, and further support the notion that NDAN represents a condition distinct from both MCI and AD. While the present results provide new evidence linking sustained neurogenesis to cognitive competency in humans, further studies are needed to fully characterize the involved mechanisms and evaluate their possible clinical significance.

## Methods

### Human subjects and autopsy brain tissues

Post-mortem brain tissue was obtained from the Oregon Brain Bank at Oregon Health and Science University (OHSU), in Portland, OR. Donor subjects were enrolled and clinically evaluated in studies at the NIH-sponsored Layton Aging and AD Center (ADC) at OHSU, in accordance with protocols that were approved by the OHSU Institutional Review Board. Informed consent was obtained from all participants prior to their enrollment in the studies at the ADC. Subjects were participants in brain aging studies at the ADC and received annual neurological and neuropsychological evaluations, with a clinical dementia rating (CDR) assigned by an experienced clinician. A neuropathological assessment was performed at autopsy, and in compliance with institutional review board-approved protocols. A neuropathologist scored autopsy brain tissue for amyloid plaques and neurofibrillary tangles, according to standardized CERAD criteria and Braak staging^32^. Participants were classified as Alzheimer’s disease (AD) when possessing a National Institute for Neurological and Communicative Disorders and Stroke-Alzheimer’s Disease and Related Disorder Association diagnostic criteria for clinical AD (CDR) including a mini-mental state exam (MMSE) score below 10. Control participants performed normally in cognitive examinations (MMSE of 28–30). Mild cognitive impairment (MCI) cases had some cognitive impairment with MMSE scores in the mid 20 s. Non-demented with AD pathology (NDAN) cases displayed little to no cognitive impairment (MMSE 26–30) while having extensive amyloid plaques and neurofibrillary tangles comparable to fully symptomatic AD (Table 1). Donor subject samples were de-identified by ADC prior to being provided to UTMB, so that no approval was required from the UTMB Institutional Review Board under CFR §46.101(a)(1).

Of note, the majority of subject’s brains used for this study were from female donors (of the 17 subjects’ brain analyzed only 3 were males). This gender make-up is not driven by any peculiar factor in our cohort (being similar in all groups studied), but likely a reflection of the fact that women have a longer life expectancy than men (81.4 years for women and 76.4 years for men)^33^.

### Tissue processing and immunofluorescence

Fresh frozen hippocampal tissue blocks were removed from storage at −80 °C, embedded in O.C.T. compound (Tissue-Tek; Tokyo, Japan) and sectioned at 10 μm onto Superfrost/Plus slides (Fisherbrand; Fisher Scientific, U.S.A.). Prepared slides were stored at −80 °C until use. Slides were removed from −80 °C and fixed in 4% paraformaldehyde in 0.1 M PBS, pH 7.4 for 15 minutes at room temperature, permeabilized with 5% normal goat serum (NGS)/0.3% Triton X-100/0.05% Tween-20, and incubated with primary antibodies diluted in PBS containing 1.5% NGS overnight at room temperature. Primary antibodies used were rabbit anti-SOX2 (1:200; Cell Signaling, Danvers, MA) and mouse anti-NeuN (1:1000; Millipore, Billerica, MA). Slides were washed in PBS before incubation with Alexa-conjugated secondary antibodies (donkey-anti rabbit and donkey anti-goat; 1:400; Life Technologies, USA) in PBS containing 1.5% NGS for 1 hour at room temperature. Finally, slides were washed in PBS, treated with 0.3% Sudan Black B (in 70% EtOH), washed again, and coverslipped using Vectashield mounting medium containing DAPI (Vector Laboratories, Burlingame, CA, US).

### Immunofluorescence image analysis and cell counts

The slides were imaged using a confocal scanning module (Bio-Rad Radiance 2000 with LaserSharp software, Hercules, CA, USA) and a 20X/0.75NA objective. Fluorescent images were acquired using constant settings for laser power, detector gain and offset. For each subject, 3 sections were used for quantification. For each section, three images were taken along the granular cell layer (GCL) and subgranular zone (SGZ; defined as an area adjacent to and equal in thickness to the GCL) of the dentate gyrus region. Image analysis was performed using ImageJ. An independent evaluator blind to the experimental groups counted the number of SOX2^+^ and NeuN^+^ cells in each of the 3 fields of view per sections. DAPI (4′,6-diamidino-2-phenylindole) was used to counterstain all nuclei. The number of cells positive for SOX2 only, NeuN only or both SOX2 and NeuN was calculated and expressed as percentage of the total number of DAPI-positive nuclei in the DG. The number of cells in each field was assessed by counting nuclei stained with DAPI. No significant differences were found in the total number of DAPI^+^ nuclei both within each experimental group and between the experimental groups (data not shown).

### Nuclei isolation

Nuclei were isolated from human and mouse total hippocampus tissue. All procedures were performed at 0–4 °C. The frozen hippocampal tissue was dissected on dry ice using surgical blade and further homogenized using dounce homogenizer (~20 strokes) in ice cold PBS. The homogenate was spun down at 800 g for 5 min at 4 °C and the pellet resuspended in lysis buffer containing CEB A, DTT and Phosphatase Inhibitors (Nuclear/Cytosol Fractionation Kit; Biovision, CA). The mixture was incubated for 15 min on ice followed by addition of buffer CEB B (Nuclear/Cytosol Fractionation Kit; Biovision, CA) and centrifuged at 830 g for 10 min at 4 °C. The pellet, containing nuclei, was resuspended in lysis buffer (Nuclei Isolation Kit; Sigma-Aldrich, MO) and incubated on ice for 10 min. The nuclei mixture was layered onto the 1.1 M (for human sample) and 1.8 M (for mouse sample) sucrose cushions (Nuclei Isolation Kit; Sigma-Aldrich, MO) in the ultracentrifuge tube. The samples were spun down at 30,000 g at 4 °C for 25 min to obtain transparent sediment at the bottom of the tube. The pellet was resuspended in ice cold PBS and centrifuged at 10,000 g at 4 °C for 5 min. The nuclei were stored in nuclei storage buffer (Nuclei Isolation Kit; Sigma-Aldrich, MO) at −20 °C until the day of FACS analysis.

### Immunolabeling and flow cytometry analysis of NeuN and SOX2

For flow cytometry analysis of NeuN and SOX2, the nuclei were spun down at 10,000 g at 4 °C for 5 min and resuspended in sodium citrate buffer (1 mg/ml sodium citrate, 1% Triton-X100 in Ca^2+^ and Mg^2+^ free PBS) containing RNase A (Sigma-Aldrich, MO) to prevent clumping of the nuclei. Nuclei were incubated for 1 hour at 4 °C on a rotating shaker, centrifuged at 10,000 g at 4 °C for 5 min and fixed in 4% paraformaldehyde at 4 °C for 10 min. The nuclei were then permeabilized in ice cold 100% methanol for 30 min. The nuclei were pelleted and resuspended with antibodies diluted in incubation buffer (3% BSA in PBS). Human nuclei were incubated with PE-conjugated anti-NeuN antibody (1:10; Millipore, MA) and with Alexa 647-conjugated anti-SOX2 antibody (1:50; Cell Signaling, MA). Mouse nuclei were incubated with Alexa 488-conjugated anti-NeuN antibody (1:20; Millipore, MA) and Alexa 647-conjugated anti-SOX2 antibody (1:20; BD Biosciences, NJ). Following incubations with the antibodies, nuclei were washed with incubation buffer and resuspended in PBS. Flow cytometry was performed with a Guava easyCyte (Millipore, MA) equipped with 488 nm blue laser and 640 nm red laser. Sample flow rate was ~3000 events per second; 10,000 ungated events were collected for analysis. Analysis was performed using InCyte software (Millipore, MA). As a control, background fluorescent measurement was performed for each sample and the gates were adjusted accordingly to eliminate the background fluorescence.

### Laser Capture Microdissection

Laser capture microdissection was performed as previously described^34^. Briefly, human hippocampus sections (10 μm thick) were stained using cresyl violet and dehydrated in graded alcohol solutions under RNAse-free conditions. The dentate gyrus (DG) including the granular cell layer (GCL) and subgranular zone (SGZ; defined as an area adjacent to and equal in thickness to the GCL), were captured and collected onto CapSure MacroLCM caps, using an ArcturusXT LCM system (Applied BioSciences/ThermoFisher). A total of ten sections were used per each subject. The captured cells were placed into lysis buffer from the RNAqueous-Micro Total RNA Isolation Kit (Ambion/ThermoFisher) and stored at −80 °C until RNA preparation.

### Quantitative RT-PCR of miRNAs

Total RNA was prepared using the RNAqueous kit from Ambion (Life Technologies/ThermoFisher; USA) according to the manufacturer’s instructions. Reverse transcription was performed utilizing the reagents and protocols provided with the miScriptII RT kit (Qiagen), using 10 ng total RNA for the RT reaction. Primers for qPCR were purchased from the miScript Primer Assays products (Qiagen), and used according to the manufacturer’s instructions. The primer sequences purchased were as follows: miR-9-5p (5′UCUUUGGUUAUCUAGCUGUAUGA), miR-25-3p (5′CAUUGCACUUGUCUCGGUCUGA), miR-29a-3p (5′UAGCACCAUCUGAAAUCGGUUA), miR-124-3p (5′UAAGGCACGCGGUGAAUGCC), miR-132-3p (5′UAACAGUCUACAGCCAUGGUCG), and miR-137 (5′UUAUUGCUUAAGAAUACGCGUAG).

### Statistical Analysis

Statistical analysis was performed using Sigmaplot. One-way ANOVA with Holm-Sidak pairwise multiple-comparison analysis post hoc test was used to determine statistical significance. If the data failed the normality test (Shapiro-Wilk), a Bonferroni correction was made before statistical analysis and is noted in the legend. Data is expressed as mean ± SEM.

## Additional Information

**How to cite this article**: Briley, D. *et al*. Preserved neurogenesis in non-demented individuals with AD neuropathology. *Sci. Rep.* **6**, 27812; doi: 10.1038/srep27812 (2016).

## Supplementary Material

Supplementary Information

## Figures and Tables

**Table 1:** Clinical data of the subjects used in the study.

Diagnosis	Subject#	Age(yrs)	Sex	Braak stage	MMSE	PMI(hr)
Ctrl	1104	86	F	2	29	16
Ctrl	1229	>89	F	2	30	12
Ctrl	1563	80	M	1	30	2
Ctrl	1731	74	F	2	29	7.5
MCI	781	89	F	3	22	20
MCI	811	>89	F	5	20	12
MCI	975	>89	F	2	25	4
AD	995	81	F	6	0	12
AD	1770	82	F	6	15	6.5
AD	1678	76	F	6	1	25
AD	1774	>89	M	6	2	3.3
AD	1776	>89	F	6	6	6.3
AD	1777	67	F	6	9	20.5
NDAN	1016	>89	F	6	26	8
NDAN	1317	>89	F	6	27	4.5
NDAN	1362	>89	F	4	27	48
NDAN	697	>89	M	5	29	5

Braak stage: a measure of the number and location of tau tangles and Aβ plaques in the brain. MMSE: Mini Mental State Examination (administered within the last year). PMI: Post Mortem Interval.
