# Supplementary material for: Preserved neurogenesis in non-demented individuals with AD neuropathology
Source: Sci Rep. 2016 Jun 14;6:27812. doi: 10.1038/srep27812 (PMC4906289; doi:10.1038/srep27812)
Supplement: Supplementary Information [file srep27812-s1.pdf]

Supplementary Figures

for

Preserved neurogenesis in non-demented individuals with AD neuropathology.

David Briley, Valeria Ghirardi, Randy Woltjer, Alicia Renck, Olga Zolochevska, Giulio Taglialatela, Maria-Adelaide Micci

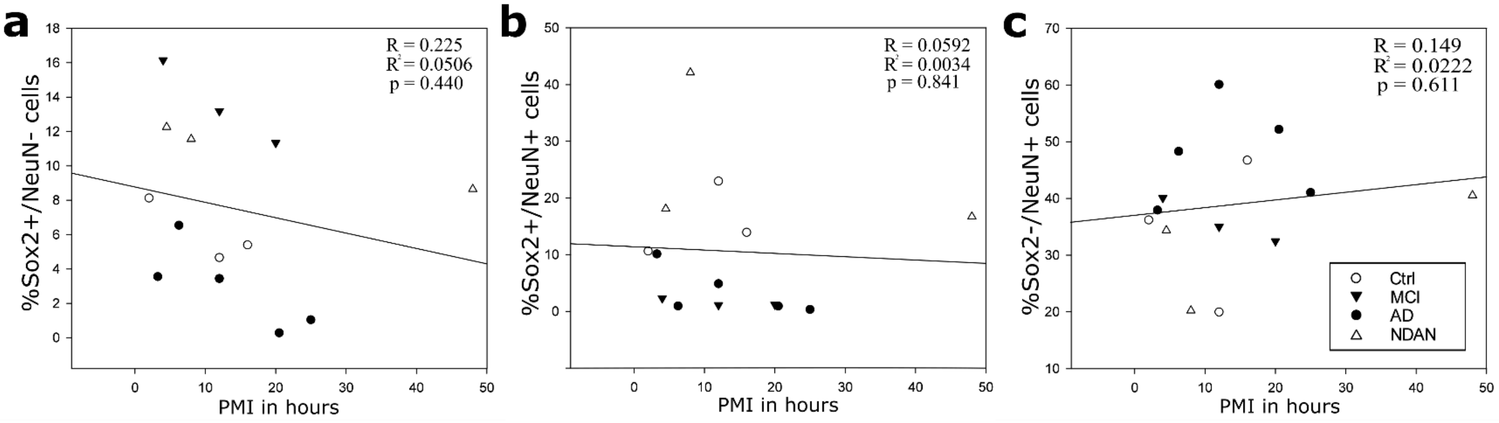

Supplementary Figure 1. The postmortem interval does not correlate with the measurements made. Correlation analysis between the postmortem interval and SOX2 expression show no significant interaction.
